# mGlu1 Receptors Monopolize the Synaptic Control of Cerebellar Purkinje Cells by Epigenetically Down-Regulating mGlu5 Receptors

**DOI:** 10.1038/s41598-018-31369-7

**Published:** 2018-09-06

**Authors:** Serena Notartomaso, Harumi Nakao, Giada Mascio, Pamela Scarselli, Milena Cannella, Cristina Zappulla, Michele Madonna, Marta Motolese, Roberto Gradini, Francesca Liberatore, Micaela Zonta, Giorgio Carmignoto, Giuseppe Battaglia, Valeria Bruno, Masahiko Watanabe, Atsu Aiba, Ferdinando Nicoletti

**Affiliations:** 1 I.R.C.C.S. Neuromed, Pozzilli, 86077 Italy; 2 Laboratory of Animal Resources, Center for Disease Biology and Integrative Medicine, Faculty of Medicine, The University of Tokyo, Bunkyo-ku, Tokyo 113-0033 Japan; 3 Department of Experimental Medicine, University Sapienza of Rome, Rome, 00185 Italy; 4 Departments of Physiology and Pharmacology, University Sapienza of Rome, Rome, 00185 Italy; 5 Institute of Neuroscience, CNR, University of Padua, Padua, 35122 Italy; 6 Department of Anatomy, Hokkaido University Graduate School of Medicine, Sapporo, 060-8638 Japan

## Abstract

In cerebellar Purkinje cells (PCs) type-1 metabotropic glutamate (mGlu1) receptors play a key role in motor learning and drive the refinement of synaptic innervation during postnatal development. The cognate mGlu5 receptor is absent in mature PCs and shows low expression levels in the adult cerebellar cortex. Here we found that mGlu5 receptors were heavily expressed by PCs in the early postnatal life, when mGlu1α receptors were barely detectable. The developmental decline of mGlu5 receptors coincided with the appearance of mGlu1α receptors in PCs, and both processes were associated with specular changes in CpG methylation in the corresponding gene promoters. It was the mGlu1 receptor that drove the elimination of mGlu5 receptors from PCs, as shown by data obtained with conditional mGlu1α receptor knockout mice and with targeted pharmacological treatments during critical developmental time windows. The suppressing activity of mGlu1 receptors on mGlu5 receptor was maintained in mature PCs, suggesting that expression of mGlu1α and mGlu5 receptors is mutually exclusive in PCs. These findings add complexity to the the finely tuned mechanisms that regulate PC biology during development and in the adult life and lay the groundwork for an in-depth analysis of the role played by mGlu5 receptors in PC maturation.

## Introduction

Group-I mGlu receptors include mGlu1 and mGlu5 receptor subtypes, which are both coupled to G_q/11_ and are predominantly found in the peripheral annulus of postsynaptic densities, where they are linked to Homer proteins and other scaffolding and effector proteins^1,2^. In spite of these similarities, mGlu1 and mGlu5 receptors differ in their anatomical distribution, with mGlu5 receptors showing a widespread expression pattern in the brain and spinal cord, and mGlu1 receptors being restricted to the olfactory bulb, thalamus, hippocampal dentate gyrus, and cerebellum^3,4^. In the cerebellum, the mGlu1 receptor is predominantly expressed in PCs, where it plays a crucial role in mechanisms of developmental plasticity and in the control of motor learning and motor coordination^5–7^. Activation of mGlu1 receptors in PC dendrites by the glutamate released from parallel fibers drives the elimination of suprannumerary climbing fibers between the second and third week of postnatal life through a mechanism that involves stimulation of polyphosphoinositide (PI) hydrolysis, activation of protein kinase C (PKC)-γ, and induction of semaphorine-7A^8,9^. The mGlu1 receptor is also required for the elimination of parallel fiber synapses from dendritic portions in which parallel and climbing fibers overlap, leading to the segregation of climbing and parallel fiber synaptic territories^8,10^. Genetic deletion of mGlu1 receptors causes a severe impairment of long-term depression (LTD) at parallel fiber-PC synapses associated with a profound defect in the conditioned eyeblink reflex and motor coordination. All these defects are rescued by selective re-introduction of mGlu1α receptors in PCs^11,12^. Interestingly, abnormalities in the expression/activity of mGlu1 receptors or downstream signaling molecules in PCs have been found in genetic mouse models of type-1, -2 -3, -5, and -14 spinocerebellar ataxias^13–16^, in ataxic *moonwalker* mutant mice^17^, in mice subjected to experimental autoimmune encephalomyelitis, and in autoptic samples from individuals affected by multiple sclerosis^18^. Neutralizing autoantibodies directed against mGlu1 receptors have been detected in patients with autoimmune ataxia^19–21^. All these findings suggest that mGlu1 receptors might be targeted by therapeutic intervention in cerebellar disorders^7^.

As opposed to mGlu1 receptors, mGlu5 receptors are virtually absent in mature cerebellar PCs, and show low expression levels in the adult cerebellar cortex, being mainly localized in Lugaro and Golgi cells^22,23^. This dampened the interest for the study of mGlu5 receptors in the cerebellum, although changes in cerebellar mGlu5 receptor expression were found in autoptic tissues from individuals affected by psychiatric disorders, autism, and Fragile X-associated tremor/ataxia syndrome^24–27^. We were intrigued by the finding that mGlu5 receptor protein levels in the rat cerebellum are higher in the early postnatal life than in the adult life, as opposed to mGlu1α receptor protein levels, which are more abundant in the adulthood than at PND9^28^. This opposite developmental pattern of expression of mGlu1α and mGlu5 receptors was characteristic of the cerebellum and was not observed in other brain regions including the hippocampus, corpus striatum, cerebral cortex, hypothalamus, and olfactory bulb^28^.

Where precisely the mGlu5 receptor is expressed in the developing cerebellum is unknown, at present. No evidence exists to our knowledge that developing PCs express mGlu5 receptors, although mGlu5 receptor knockdown in the early postnatal life causes a severe impairment in PC maturation^29^.

We now report that mGlu5 receptors are highly expressed by cerebellar PCs in the first 12 days of postnatal life, and that the developmental decline in mGlu5 receptor expression coincides with the appearance and up-regulation of mGlu1α receptors. In addition, we demonstrate that it is the mGlu1 receptor that down-regulates the expression of mGlu5 receptors during the development of PCs and maintains its suppressing activity in the adult life.

## Results

### Complementary expression of mGlu1α and mGlu5 receptors in developing cerebellar PCs

**Figure 1:**
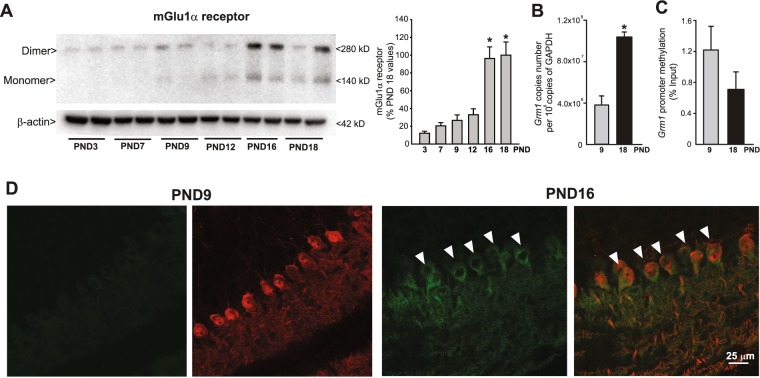
Developmental pattern of expression of mGlu1 receptors in the mouse cerebellar PCs. mGlu1α protein levels in the mouse cerebellum at different PNDs are shown in (**A**). Densitometric values are means ± S.E.M of 4–5 mice/group. *p < 0.001 (One-Way ANOVA + Fisher’s LSD; F_(5,20)_ = 21.8) *vs*. values obtained in mice at PND18. *Grm1* mRNA levels and *Grm1* promoter methylation in the cerebellum at PND9 and PND18 are shown in (**B**) and (**C**), respectively. Values are means ± S.E.M. of 5 mice/group. *p < 0.001 *vs*. values at PND9 (Student’s t test; t_(7)_ = −7.96). Confocal microscopy analysis of mGlu1α receptors (green) and calbindin (red) in the cerebellar cortex is shown in (**D**). Note the faint mGlu1α immunoreactivity in PCs at PND9. Arrowheads indicate mGlu1α receptor immunoreactivity in PCs.

We examined mGlu1α receptor expression in cerebellar tissue from mice at 3, 7, 9, 12, 16, and 18 PNDs by combining immunoblot analysis and immunofluorescent staining. mGlu1α receptor protein levels in the cerebellum were very low in the first twelve PND and increased substantially from PND12 to PND16, remaining high at later stages of development (Fig. 1A). The transcript of mGlu1 receptors increased by >2 fold from PND9 to PND18 (Fig. 1B), and this was associated with a trend to a reduction in *Grm1* promoter methylation (Fig. 1C). Confocal analysis showed that expression of mGlu1α receptors was faint in calbindin-positive PCs at PND9 and increased substantially at PND16 (Fig. 1D).

**Figure 2:**
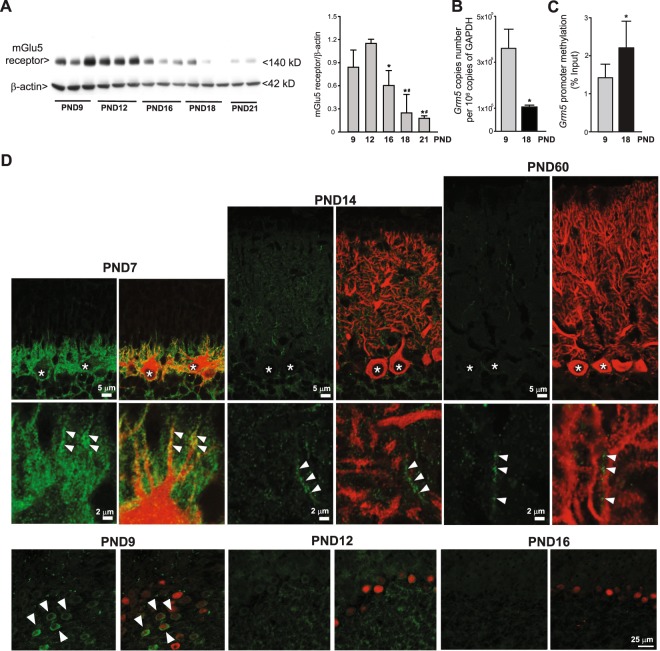
Expression of mGlu5 receptors in cerebellar PCs early after birth. Western blot analysis of mGlu5 receptors in the mouse cerebellum at different PNDs is shown in (**A**). Densitometric values are means ± S.E.M. of 2–3 mice/group. p = 0.008 *vs*. values at PND9 (#) or PND12 (*) (One-Way ANOVA + Fisher’s LSD; F_(4,9)_ = 7.01). The β-actin was taken from the same blot but with a lower exposure time. *Grm5* mRNA levels and *Grm5* promoter methylation in the cerebellum at PND9 and PND18 are shown in (**B**) and (**C**), respectively. Values are means ± S.E.M. of 4 mice/group. *p = 0.04 *vs*. values at PND9 (Student’s t test; t_(6)_ = 2.61), *p = 0.006 *vs*. values at PND9 (Student’s t test; t_(6)_ = −4.11), for *Grm5* mRNA levels and *Grm5* promoter methylation, respectively. Confocal microscopy images of mGlu5 receptor immunoreactivity in PCs at different PNDs are shown in D. Images were obtained from two different experiments performed in different laboratories. In the upper panel mGlu5 receptors are in green and type-8 carbonic anhydrase (a marker of PCs) is in red. In the lower panel, mGlu5 receptors are in green and calbindin (another marker of PCs) is in red. Note the disappearance of mGlu5 receptors in PCs after PND12. * indicates PCs; arrowheads indicate mGlu5 receptor immunoreactivity in PCs.

Interestingly, the mGlu5 receptor showed a complementary pattern of expression in the developing cerebellum, with mGlu5 receptor protein levels being high between PND9 and PND12 and decreasing afterwards (Fig. 2A). The transcript of mGlu5 receptors was dramatically reduced from PND9 to PND18 (Fig. 2B), and this was associated with a significant increase in *Grm5* gene promoter methylation (Fig. 2C). Confocal microscopy analysis of mGlu5 receptors was performed in two separate experiments at different time points after birth. Developing PCs labelled for carbonic anhydrase-8 (Fig. 2D, upper panel) or calbindin (Fig. 2D, lower panel) expressed mGlu5 receptors at PND7, 9, and 12, but were no longer decorated with mGlu5 receptor antibodies after PND14 (Fig. 2D). After PND14 weak labelling for mGlu5 receptors remained in carbonic anhydrase-8 negative dendritic elements of putative interneurons.

### Developmental switch from mGlu1 and mGlu5 receptor signaling in the postnatal cerebellum

**Figure 3:**
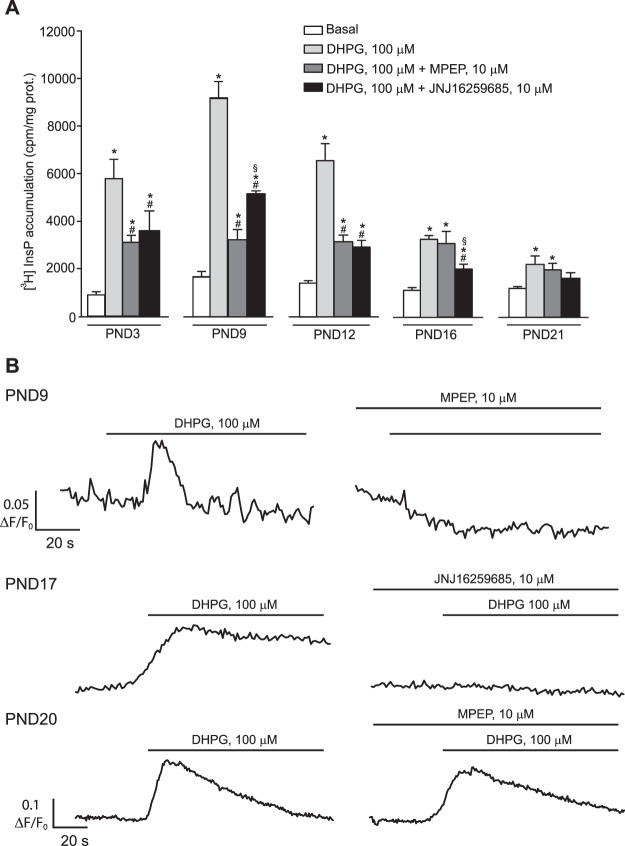
Developmental pattern of mGlu1 and mGlu5 receptor signaling in the cerebellum and cerebellar PCs. DHPG-stimulated PI hydrolysis in cerebellar slices at different PNDs incubated in the absence or presence of MPEP or JNJ16259685 is shown in (**A**). Values are means ± S.E.M. of 3–5 determinations. p < 0.001 *vs*. the respective basal values (*), *vs*. the respective values obtained with DHPG alone (#), or *vs*. the respective values obtained with DHPG in combination with MPEP ($) (One-Way ANOVA + Fisher’s LSD; PND3: F_(3,8)_ = 16.74; PND9: F_(3,13)_ = 59.93; PND12: F_(3,12)_ = 37.07; PND16: F_(3,12)_ = 15.97; PND21: F_(3,12)_ = 3.79). DHPG-stimulated intracellular Ca^2+^ release in cerebellar PCs at different PNDs is shown in (**B**). Note that MPEP antagonized the action of DHPG at PND9 but was ineffective at PND20. In contrast, the action of DHPG was antagonized by JNJ16259685 at PND17.

We used cerebellar slices challenged with the mGlu1/5 receptor agonist, DHPG, in the presence of mGlu1 or mGlu5 receptor blockers for the analysis of receptor-stimulated PI hydrolysis. As expected, maximally effective concentrations of DHPG (100 μM) largely increased [^3^H]inositolmonophosphate (InsP) formation in PND3-12 cerebellar slices. Stimulation of PI hydrolysis decreased at PND16 and became very low at PND21 (Fig. 3A). In the first 12 days of postnatal life, DHPG-stimulated PI hydrolysis was highly sensitive to the mGlu5 receptor negative allosteric modulator (NAM), MPEP, whereas only the mGlu1 receptor NAM, JNJ16259685, was able to antagonize the action of DHPG at PND16 (Fig. 3A). To localize mGlu1 or mGlu5 receptor signaling at cellular level, we assessed intracellular Ca^2+^ release in PCs from cerebellar slices prepared from PND9, PND17, and PND20. At PND9, DHPG-stimulated Ca^2+^ release was abrogated by MPEP (Fig. 3B). In contrast, the action of DHPG was antagonized by JNJ16259685, but not by MPEP, at PND17 and PND20 (Fig. 3B).

### mGlu1 receptors drive the developmental decline of mGlu5 receptors in PCs

**Figure 4:**
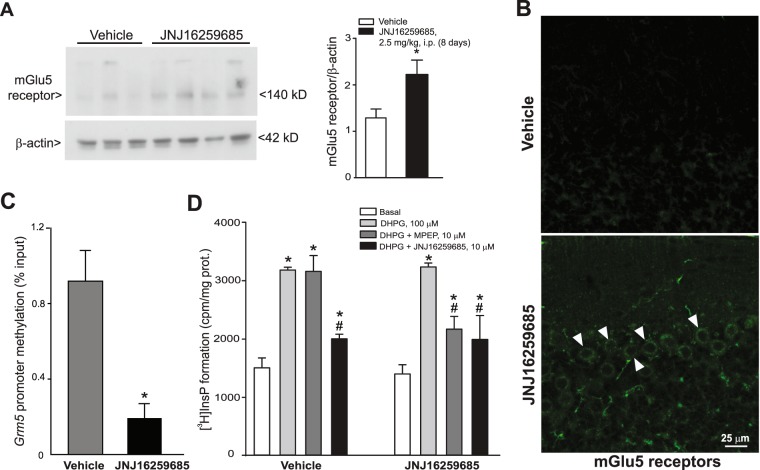
Pharmacological blockade of mGlu1 receptors delayed the developmental decline in mGlu5 receptors in cerebellar PCs. Mice were treated daily with JNJ16259685 (2.5 mg/kg, i.p.) or its vehicle from PND9 to PND16. All measurements were carried out at the end of treatments. Western blot analysis of mGlu5 receptors in the cerebellum is shown in (**A**). Densitometric values are means ± S.E.M. *p = 0.041, *vs*. mice treated with vehicle (Student’s t test; t_(5)_ = −2.74). Confocal microscopy analysis of mGlu5 receptors (green) is shown in (**B**). Note that mGlu5 receptor immunoreactivity is still present in PCs at PND16 only in mice treated with JNJ16259685. *Grm5* promoter methylation in mice treated with vehicle or JNJ16259685 is shown in (**C**), where values are means ± S.E.M. of 3 mice/group. *p = 0.0076 *vs*. values obtained in mice treated with vehicle (Student’s t test; t_(4)_ = 4.98). DHPG-stimulated PI hydrolysis in cerebellar slices from mice treated with vehicle or JNJ169259685 is shown in (**D**). Note that MPEP maintained its antagonistic activity in slices from PND16 mice treated with JNJ16259685. Values are means ± S.E.M. of 4 mice/group. (*) *vs*. the respective basal values or (#) *vs*. the respective values obtained with DHPG alone (Two-Way ANOVA + Fisher’s LSD; mouse treatment: F_(1,24)_ = 7.13, p = 0.013; slice treatment: F_(3,24)_ = 59.42, p < 0.001; mouse treatment x slice treatment: F_(3,24)_ = 6.05, p = 0.003). Arrowheads indicate mGlu5 receptor immunoreactivity in PCs.

To examine whether the decline of mGlu5 receptors in PCs was causally related to the appearance of mGlu1α receptors during postnatal development, we used pharmacological and genetic approaches. In a first set of experiments, we treated mice daily with the mGlu1 receptor NAM, JNJ16259685 (2.5 mg/kg, i.p.) during a developmental period that corresponds to the appearance of mGlu1 receptors, i.e., from PND9 to PND16. Pharmacological blockade of mGlu1 receptors during this time window prevented the decline of mGlu5 receptor protein, which was still expressed in PCs at PND16 (Fig. 4A,B). *Grm5* gene promoter methylation was reduced in the cerebellum after treatment with JNJ16259685 (Fig. 4C). In addition, MPEP retained the activity of antagonizing DHPG-stimulated PI hydrolysis in cerebellar slices after *in vivo* treatment with JNJ16259685 (Fig. 4D).

**Figure 5:**
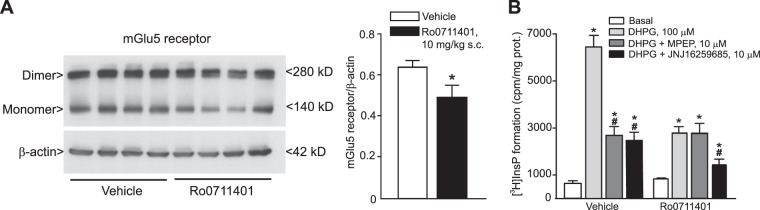
Pharmacological enhancement of mGlu1 receptors accelerates the developmental decline of mGlu5 receptors in the cerebellum. Mice were treated with Ro0711401 (10 mg/kg, s.c., daily) or its vehicle from PND7 to PND12 in (**A**) or from PND10 to PND12 in (**B**). Measurements were carried out at the end of the treatment. Western blot analysis of mGlu5 receptors in the cerebellum is shown in (**A**). Densitometric values are means ± S.E.M. of 4 mice/group. *p = 0.042 *vs*. values obtained in mice treated with vehicle (Student’s t test; t_(6)_ = 2.57). DHPG-stimulated PI hydrolysis in cerebellar slices prepared from PND12 mice treated with vehicle or Ro0711401 is shown in (**B**). Note that MPEP lost its antagonistic activity in slices from mice treated with Ro1711401. Values are means ± S.E.M. of 4 mice/group. (*) *vs*. the respective basal values or *vs*. (#) or vs. the respective values obtained with DHPG alone. (Two-Way ANOVA + Fisher’s LSD; mouse treatment: F_(1,24)_ = 38.46, p < 0.001; slice treatment: F_(3,24)_ = 78.76, p < 0.001; mouse treatment x slice treatment: F_(3,24)_ = 20.59, p < 0.001).

Alternatively, we treated mice with the selective mGlu1 receptor positive allosteric modulator (PAM), Ro0711401 (10 mg/kg, s.c., daily), from PND7 (for Western blot analysis) or PND10 (for functional analysis) to PND12, when expression of mGlu1 receptors in PCs is still low. This treatment accelerated the drop in mGlu5 receptor protein in the cerebellum, which was already reduced at PND12 (Fig. 5A). When DHPG-stimulated PI hydrolysis was monitored in cerebellar slices, MPEP lost its antagonistic activity at PND12 after systemic treatment with Ro0711401 (Fig. 5B). These data suggested that endogenous activation of mGlu1 receptors was responsible for the developmental drop of mGlu5 receptors in PCs.

**Figure 6:**
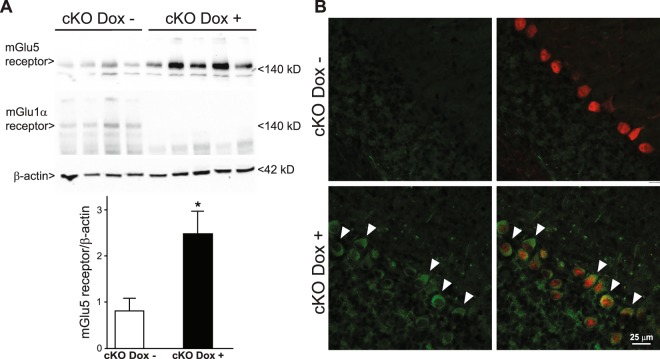
mGlu1 receptors drive the developmental decline of mGlu5 receptors in PCs. Conditional mGlu1α receptor knockout mice (cKO mice) were treated with doxycycline (200 μg/ml in drinking water between PND0 to PND14). Western blot analysis of mGlu1α and mGlu5 receptors in the cerebellum at PND16 is shown in (**A**). Densitometric values are means ± S.E.M. of 4–5 mice/group. *p = 0.017 *vs*. mGlu1α cKO mice not treated with Doxycycline (Dox−) (Student’s t test; t_(7)_ = −3.10). Confocal microscopy analysis of mGlu5 receptors (green) and calbindin (red) is shown in (**B**). Arrowheads indicate mGlu5 receptor immunoreactivity in PCs.

To further demonstrate the developmental link between mGlu1 and mGlu5 receptors in PCs, we used conditional mGlu1 receptor knockout (cKO) mice. In these mice, exposure to doxycyclin (added to the drinking water of lactating mothers) from PND0 to PND14 suppressed the expression of mGlu1 receptors in PCs of the offspring (Fig. 6A). Doxycylin-induced knockdown of mGlu1 receptors was associated with a large increase in mGlu5 receptor expression, which was still prominent in the PCs at PND16 (Fig. 6A,B).

### mGlu1 receptors retain the suppressive activity on mGlu5 receptor expression in adult PCs

**Figure 7:**
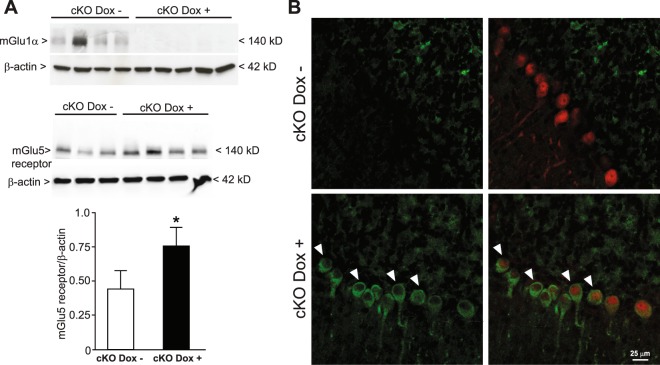
mGlu1 receptors retain the suppressive activity on mGlu5 receptor expression in adult PCs. Conditional mGlu1α receptor knockout mice (cKO mice) were treated with doxycycline (200 μg/ml in drinking water between PND24 to PND76). Western blot analysis of mGlu1α and mGlu5 receptors in the cerebellum is shown in (**A**). Densitometric values are means ± S.E.M. of 3–4 mice per group. *p = 0.026 *vs*. mGlu1α cKO mice not treated with Doxycycline (Dox−) (Student’s t test; t_(5)_ = −3.12). Confocal microscopy analysis of mGlu5 receptors (green) and calbindin (red) is shown in (**B**). Arrowheads indicate mGlu5 receptor immunoreactivity in PCs.

mGlu1α receptor cKO mice were also used to examine whether mGlu1 receptors could maintain the mGlu5 receptor suppressed in PCs also in the life. Chronic treatment with doxycyclin (200 μg/ml in the drinking water) from PND24 to PND76 caused the expected suppression of mGlu1α receptor protein associated with a significant increase in mGlu5 receptor protein levels in the cerebellum (Fig. 7A). Remarkably, cerebellar PCs were highly decorated with mGlu5 receptors after chronic mGlu1α receptor knockdown (Fig. 7B).

## Discussion

Our findings demonstrate for the first time that mGlu5 receptors are physiologically expressed by cerebellar PCs in the early stages of postnatal development, and largely mediate group-I mGlu receptor-stimulated PI hydrolysis in the first two weeks of postnatal life. Interestingly, the developmental pattern of expression of mGlu1α and mGlu5 receptors in PCs was specular, with mGlu1α receptors entirely replacing mGlu5 receptors during the first two weeks after birth. Gene methylation analysis demonstrated that the developmental switch between mGlu5 and mGlu1 receptors was driven by an epigenetic mechanism. Of particular interest was the demonstration that the appearance of mGlu1α receptors caused the developmental decline of mGlu5 receptors in PCs. Pharmacological experiments with mGlu1 receptor ligands administered to mice in restricted developmental time windows suggested that endogenous activation of mGlu1 receptors restrained mGlu5 receptor expression by enhancing *Grm5* promoter methylation.

These findings raise a number of fundamental questions: (i) why is the mGlu5 receptor highly expressed by PCs in the first twelve days of postnatal life?; (ii) why a robust stimulation of PI hydrolysis is needed during that period?; (iii) why the mGlu1 receptor monopolizes the control of PC physiology by eliminating the mGlu5 receptor after the first two weeks of postnatal life?; and, (iv) are the two receptors different considering that they belong to the same subgroup and are both coupled to PI hydrolysis as a primary signal transduction mechanism?

Hydrolysis of phosphatidylinositol-4,5-bisphosphate generates inositol-1,4,5-trisphosphate (InsP_3_) and dyacylglycerol (DAG), which stimulates intracellular Ca^2+^ release and activates protein kinase C (PKC), respectively^30^. Experiments carried out in heterologous expression systems have shown that mGlu1 and mGlu5 receptors differ in the kinetics of intracellular Ca^2+^ release in response to receptor activation. Glutamate elicits single-peaked intracellular Ca^2+^ mobilization in cells expressing mGlu1 receptors and induces Ca^2+^ oscillations in cells expressing mGlu5 receptors^31^. This difference relies on a threonine residue of mGlu5 receptor (T845) that is localized in the G-protein interaction domain and is phosphorylated by PKC^31^. Although PCs are generated before birth, they undergo dramatic morphological changes in a postnatal time window that corresponds to mGlu5 receptor expression, particularly after PND7 when PCs develop their typical dendritic trees^32^. Dendritic development in rodent PCs proceeds through an initial phase in which the primitive dendritic tree is retracted and multiple filopodia-like processes are formed around the cell body (see Fig. 2D at PND7). After PND7, these processes disappear and the final dendritic tree starts to grow. This second phase of dendritic growth is driven by both intrinsic and extrinsic factors, including the glutamate release from afferent fibers^33^. Interestingly, a phase of developmental PC death precedes the onset of the second phase of dendritic growth^33^. Activation of mGlu5 receptors with ensuing stimulation of PI hydrolysis and intracellular Ca^2+^ oscillations might have a key role in any of these early postnatal events and might provide a survival signal for those Purkinje neurons that undergo early innervation by climbing and parallel fiber. Activation of mGlu5 receptors might also be involved in the early elimination phase of supranumerary climbing fibers (from PND7 to PND15), the underlying mechanism of which is still unknown^7,34^.

Activation of mGlu1 receptors after PND12 might provide a more localized Ca^2+^ signal, which, together with PKC-γ activation might be instrumental for the late phase of elimination of supranumerary climbing fibers *via* the induction of semaphorin-7A or other mechanisms^9^, as well as for parallel fiber elimination and territory segregation to the distal dendrites of PCs occurring after PND15^10^. mGlu1 receptors might optimize the signal-to-noise ratio in intracellular Ca^2+^ release during an active period of synaptic refinement by eliminating a competitor receptor (the mGlu5 receptor) that generates Ca^2+^ oscillations and waves that may spread across the cytoplasm. In addition, elimination of mGlu5 receptors might serve to deprive PCs of a morphogenetic signal that becomes inappropriate during a period of active synaptic refinement.

Interestingly, mGlu1 receptors maintained the suppressive action on mGlu5 receptors in the adult life, as shown by the reappearance of mGlu5 receptors in PCs after genetic deletion of mGlu1α receptors. At least four functional splice variants of mGlu1 receptors (named mGlu1α or a, -β1 or b, -β2 or f, and -γ or d) have been described^35^, of which mGlu1α predominates in the cerebellum, and mGlu1α and mGlu1β1 are expressed in PCs^36,37^. The long C-terminal domain exclusive of mGlu1α receptors allows interaction with Homer proteins^38^ and better phospholipase-C coupling efficacy^39,40^ and is also required for perisynaptic targeting of mGlu1 receptors, inositol-1,4,5-trisphosphate-mediated Ca^2+^ mobilization, elimination of supranumerary climbing fibers, LTD, and motor learning^37^. We showed that epigenetic regulation of mGlu5 receptor expression is dependent on mGlu1α variant. The role of mGlu1β1 receptor in reguration of mGlu5 receptor would be elucidated by use of mice in which mGlu1β1 transgene is introduced in PCs of mGlu1 receptor knockout mice.

A re-expression of mGlu5 receptors in PCs has been shown in pathological conditions characterized by a reduced expression or activity of mGlu1 receptors in PCs, i.e., in mice modelling type-1 spinocerebellar ataxia (SCA1)^15^, in mice developing experimental autoimmune encephalomyelitis (EAE)^18^, and in autoptic cerebellar samples from patients affected by multiple sclerosis^18^. This is one of the several examples of developmental proteins that are re-expressed in degenerating neurons. At least in SCA1 and EAE mice systemic treatment with mGlu5 receptor PAMs or NAMs did not affect cerebellar motor symptoms, suggesting that re-expressed mGlu5 receptors do not substitute for mGlu1 receptors in the regulation of motor learning and motor coordination. However, we cannot exclude that re-expressed mGlu5 receptors support the survival of degenerating PCs. If so, continuous treatment with a selective mGlu5 receptor PAM could slow the progression of cerebellar disorders by amplifying the otherwise sterile pro-survival program driven by mGlu5 receptors. This interesting hypothesis warrants further investigation.

## Materials and Methods

9H-Xanthene-9-carboxylic acid (4-trifluoromethyl-oxazol-2-yl)-amide (RO0711401) was kindly provided by Roche Laboratories (Basel, Switzerland); 2-methyl-6-(phenylethynyl)-pyridine (MPEP), (3,4-dihydro-2H-pyrano[2,3-b]quinolin-7-yl) (cis-4-methoxycyclohexyl) methanone (JNJ16259685), N-cyclobutyl-6-[2-(3-fluorophenyl) ethynyl]-3-pyridine-carboxamide hydrochloride (VU0360172), and (RS)-3,5-dihydoxyphenylglycine (DHPG) were purchased from Tocris Biosciences (Bristol, United Kingdom).

### Animals

All mice were kept under environmentally controlled conditions (ambient temperature, 22 °C; humidity, 40%) on a 12 h light/dark cycle with food and water ad libitum. All experiments were carried out according to the European (86/609/EEC) and Italian (D:Lgs. 116/92) guidelines of animal care and the animal welfare committees of the University of Tokyo (P14/117). The experimental protocol was approved by the Italian Ministry of Health (D.M.509/2015-PR).

C57BL/6 male mouse pups were used at postnatal day (PND) 3, 9, 12, 16, 18, 21, the day of birth was designed as PND zero.

Generation of mGlu1^−/−^ mice bearing L7-tTA and TRE-mGlu1α transgenes (mGlu1α receptor cKO mice; C57BL/6 N background) proceeded as described previously^41^. mGlu1α receptor cKO mice were untreated or treated with 200 µg/ml of Dox (doxycycline hyclate; Sigma, St Louis, MO) in drinking water between 0 and 14 days of age or between 24 and 76 days of age. The Dox-water was delivered in dark bottles to protect Dox from light and changed twice a week. At 16 or 76 days of age, brain samples of these mGlu1α receptor cKO mice were collected. For immunohistochemical analysis, these mice were perfused with 4% paraformaldehyde in 0.1 M phosphate buffer.

### RNA isolation, reverse transcription and quantitative real-time PCR

Total RNA from the cerebellum was isolated using the TRIzol reagent (Life Technologies, Monza, Italy) according to the manufacturer’s protocol and retrotranscribed into cDNA by using SuperScript III Reverse Transcriptase (Life Technologies). Real-Time PCR was performed on 20 ng of cDNA by using specific primers and Power SYBR Green Master Mix (Applied Biosystem, Foster City, CA) on an Applied Biosystems Step-One instrument. Thermal cycler conditions were as follows: 10 min at 95 °C, 40 cycles of denaturation (15 sec at 95 °C), and combined annealing/extension (1 min at 60 °C). Real-time PCR was performed by using the following primers:

mGlu1α/β receptors: forward, 5′-CATACGGAAAGGGGAAGTGA-3′; reverse, 5′-AAAAGGCGATGGCTATGATG-3′. mGlu5 receptors: forward, 5′-ACGAAGACCAACCGTATTGC-3′; reverse, 5′-AGACTTCTCGGATGCTTGGA-3′. GAPDH: forward, 5′-CGTCCCGTAGACAAAATGGT-3′; reverse, 5′-TCAATGAAGGGGTCGTTGAT-3′. mRNA levels were calculated from serially diluted standard curves simultaneously amplified with the samples and normalized to GAPDH mRNA levels.

### Methylated DNA immunoprecipitation (MeDIP) Analysis

Mouse monoclonal 5-methylcytosine (5-mC) (Diagenode # C15200081-100, lot. GF-003) antibody were used for the immunoprecipitation. Genomic DNA was extracted from mice cerebellum and sonicated, by using Covaris S220 Ultrasonicator, to produce a fragment size of 300–600 bp. After ethanol precipitation 5 μg of sonicated DNA were diluted to 300 µl in TE buffer and heat-denatured at 95 °C for 10 min. Then, 30 μl of sonicated solution were removed and stored at −20 °C to be used to quantify the total amount of promoter before immunoprecipitation (input). The remaining solution was incubated over night at 4 °C with (5-mC monoclonal antibody, 0.5 μl/IP); magnetic beads conjugated to anti-mouse-IgG were used to bind the anti-5mC antibodies, and unbound DNA was removed in the supernatant. The immunoprecipitated DNA was released from the antibody complex by proteinase-K digestion for 2 h at 50 °C. After phenol-chloroform extraction and ethanol precipitation the DNA pellet was resuspended in 20 μl of water. CpG-rich *Grm5* and *Grm1* gene promoters were measured by Q-PCR with Power SYBR Green Master Mix (Applied Biosystem) on an Applied Biosystems Step-One instrument. Thermal cycler conditions were as follows: 95 °C for 10 min, 42 cycles of (95 °C for 15 seconds, 60 °C for 1 min). Q-PCR was performed by using the following primers: Grm5promoter: forward, 5′-ACCTGCTCTCCAGCTTCTCT-3′ reverse, 5′-GCCTCTTGGTCTCAGGGTTC-3′ Grm1 promoter: forward, 5′-ATGGCCTCCACTCTCTGGAT-3′ reverse 5′-ATCGGAGCCCTCTTCTCAGT-3′. Methylation level was calculated by using the formula % (MeDNA-IP/Total input) = 2^[(Ct(20%input) − 3.32) − Ct (MeDNA-IP)]^ × 100%. Methylation level was confirmed by analysis of methylation enrichment of two promoters, unmethylated and methylated CpG island promoter genes GAPDH and histone H2B type 1-A (TSH2B) (Diagenode Cat# pp-1045-500 and #pp-1042-500).

### Western blot analysis

The cerebella were dissected out and homogenized at 4 °C in RIPA buffer containing protease inhibitors cocktail (Merck Millipore, Milano, Italy) for 30 min and an aliquot was used for protein determination.

Equal amounts of proteins (20 μg) from supernatants were separated by 8% SDS polyacrilamide gel at 100 V for 1 hour for the detection of mGlu1α receptors or mGlu5 receptors, using a mini-gel apparatus (Bio-Rad Mini Protean II cell, Milano, Italy). Proteins were than electroblotted on Immuno PVDF membranes (Bio-Rad) for 7 min using Trans Blot Turbo System (Bio-Rad). Filters were washed three times and blocked for 1 hour in Tris-Tween buffered saline (TTBS) containing 5% non-fat dry milk.

The following primary antibodies were used: mouse polyclonal anti-mGlu1α receptor antibody (1:7000, BD Biosciences Milan, Italy, Cat #556389, lot. 3172791); rabbit monoclonal anti-mGlu5 receptor antibody (1:5000, Abcam, Cambridge, UK, Cat #AB76316, lot. GR45647-18). Filters were washed three times with TTBS buffer and then incubated for 1 hour with secondary peroxidase-coupled anti-mouse antibody (1:7000, Millipore Cat #401215) and anti-rabbit antibody (1:7000, Millipore, Cat #401393-2 ML).

Immunostaining was revealed by enhanced chemiluminescence luminosity (Amersham Pharmacia Biotech, Arlington Height, IL). The blots were re-probed with monoclonal anti β-actin antibody (1:50000, Sigma-Aldrich, Cat # A5441, lot. 116M4801V).

### Confocal microscopy analysis

Mice were deeply anesthetized and perfused with ice-cold 4% paraformaldehyde, brains were dissected out and equilibrated with 30% sucrose overnight. Cerebellum was sectioned using a Leica cryostat (CM3050). For immunofluorescence analysis, serial sections were incubated with blocking solution (5% normal serum in 0.3% Triton X-100 in PBS) and then with the following antibodies overnight at 4 °C: anti-calbindin (1:500, Abcam, Cat #ab82812, lot. GR110079, and Santa Cruz Biotechnology, Dallas, TX, Cat #sc-7691, lot. G2512), anti-mGlu5 receptor (1:200, Abcam, Cat #AB76316, lot. GR45647-18) and anti-mGlu1α receptor (1:100, BD Biosciences, Cat #556389, lot. 3172791). Sections were also incubated with anti-mGlu5 receptor^42^ (1 μg/ml) and anti-type-8 carbonic anhydrase^43^ (1 μg/ml). After washing, sections were incubated with secondary antibodies conjugated with Alexa Fluor 488 (1:200, Molecular Probes, Eugene, OR, Cat #S32354) and Cy3 (1:200, Jackson ImmunoResearch Labs, West Grove, PA, Cat #715-167-003) for 2 hours at room temperature and rinsed in PBS. Finally, sections were mounted with anti-fading agent (Vector Laboratories, Burlingame, CA) and examined with ZEISS 780 confocal laser scanning microscope. We used a 488 nm argon laser to excite alexa488 and 543 HeNe laser to excite Cy3.

### Measurement of polyphosphoinositide hydrolysis in cerebellum slices

Group I mGlu receptor-stimulated polyphosphoinositide (PI) hydrolysis was measured in cerebellum slices obtained from PND 3, 9, 12, 16 and 21 mice as described previously^44^. Briefly, cerebella were dissected aut and sliced (350 × 350 μm) using a McIlwain tissue chopper (Mickle Laboratory Engineering, Guildford, UK) and transferred in Krebs/Henseleit buffer (equilibrated with 95% O_2_, 5% CO_2_ to pH 7.4). Forty μl of gravity-packed slices were then incubated for 60 min in 350 μl of Krebs/Henseleit buffer containing 1 μCi of *myo*-[^3^H]inositol (18 Ci/mmol, GE Healthcare, Milano, Italy). Slices were incubated with LiCl (10 mM for 10 min) and, after 15 min, slices were challenged with the mGlu1/5 receptor agonist, DHPG, and JNJ16259685 or MPEP, if present, were added 5 min prior to DHPG.

One hour later, the incubation was stopped by the addition of 900 μl of methanol/chloroform (2:1). After further addition of 300 μl of chloroform and 600 μl of water, samples were centrifuged at low speed to facilitate phase separation, and the upper aqueous phase was loaded into Dowex 1-X-8 columns resin (100–200 mesh, formate form; Dow Chemical Company, Midland, MI) for the separation and quantification of [^3^H]InsP.

Columns were washed twice with water, once with a solution of 5 mM sodium tetraborate and 40 mM sodium formate to elute cyclic InsP and glycerophosphoinositols, and then with 6.5 ml of 0.2 M ammonium formate and 0.1 M formic acid for the elution of [^3^H]InsP. The remaining aqueous phase and the organic phase were dried under a continuous nitrogen stream, and 0.5 N NaOH was added to each sample.

### Calcium imaging analysis

Parasagittal slices from the cerebellum were prepared from C57BL/6J mice at PND9, 17 and 20.

Briefly, animals were anesthetized, the brain including cerebellum dissected out and transferred into an ice-cold solution (artificial cerebro-spinal fluid, ACSF, in mM: 125 NaCl, 2.5 KCl, 2 CaCl_2_, 1 MgCl_2_, 25 glucose, pH 7.4 with 95% O_2_ and 5% CO_2_). Parasagittal slices (320 μm) were cut from the vermis with a Leica vibratome VT1000S, in an ice-cold solution containing the following (in mM): 130 K-gluconate, 15 KCl, 0.05 EGTA, 20 HEPES, and 25 glucose, with pH adjusted to 7.4 by NaOH, and then kept for 1 min in the solution (in mM): 225 D-mannitol, 2.5 KCl, 1.25 NaH_2_PO_4_, 26 NaHCO_3_, 25 glucose, 0.8 CaCl_2_, 8 MgCl_2_, with 95% O_2_ and 5% CO_2_.

Slices were recovered in ACSF for 30 min at 34 °C, then loaded for 45 min with the green fluorescent calcium dye OGB1-AM (10 μM) in the presence of 0.02% pluronic, and finally recovered for 45 min at room temperature.

For calcium imaging experiments, slices were perfused in a submerged chamber at a rate of 3–4 ml min-1 with (in mM): 120 NaCl, 2.5 KCl, 1 NaH_2_PO_4_, 26 NaHCO_3_, 1 MgCl_2_, 2 CaCl_2_, 10 glucose, pH 7.4 (with 95% O_2_ and 5% CO_2_). Oregon Green-loaded Purkinje neurons were visualized upon 488 nm laser excitation under a confocal microscope (TCS-SP5-RS, Leica Microsystems, Germany), and monitored at 1 Hz upon application of mGlu receptor ligands.
